# TiO_2_ Nanotubes Promote Osteogenic Differentiation Through Regulation of Yap and Piezo1

**DOI:** 10.3389/fbioe.2022.872088

**Published:** 2022-04-07

**Authors:** Keyu Kong, Yongyun Chang, Yi Hu, Hua Qiao, Chen Zhao, Kewei Rong, Pu Zhang, Jingwei Zhang, Zanjing Zhai, Huiwu Li

**Affiliations:** Shanghai Key Laboratory of Orthopaedic Implants, Department of Orthopaedic Surgery, Shanghai Ninth People’s Hospital, Shanghai Jiaotong University School of Medicine, Shanghai, China

**Keywords:** mesenchymal stem cells, osteogenic differentiation, titanium nanotubes, hippo pathway, Piezo1

## Abstract

Surface modification of titanium has been a hot topic to promote bone integration between implants and bone tissue. Titanium dioxide nanotubes fabricated on the surface of titanium by anodic oxidation have been a mature scheme that has shown to promote osteogenesis *in vitro*. However, mechanisms behind such a phenomenon remain elusive. In this study, we verified the enhanced osteogenesis of BMSCs on nanotopographic titanium *in vitro* and proved its effect *in vivo* by constructing a bone defect model in rats. In addition, the role of the mechanosensitive molecule Yap is studied in this research by the application of the Yap inhibitor verteporfin and knockdown/overexpression of Yap in MC3T3-E1 cells. Piezo1 is a mechanosensitive ion channel discovered in recent years and found to be elemental in bone metabolism. In our study, we preliminarily figured out the regulatory relationship between Yap and Piezo1 and proved Piezo1 as a downstream effector of Yap and nanotube-stimulated osteogenesis. In conclusion, this research proved that nanotopography promoted osteogenesis by increasing nuclear localization of Yap and activating the expression of Piezo1 downstream.

## Introduction

In recent years, the number of hip and knee arthroplasty procedures has increased rapidly (Schwartz et al., 2016). Application of artificial prosthesis has greatly improved the quality of patients’ lives, but its long-term complications will make patients suffer the risk of revision. Among all the causes of revision, the most common one is aseptic loosening (Kahlenberg et al., 2019; Kelmer et al., 2021). Due to unsatisfactory interface bone integration between the prosthesis and bone tissue, there would be loosening between prosthesis and bone tissue in the long-term fretting environment. Therefore, how to enhance bone integration between prosthesis and bone tissue has always been a hot topic (Shah et al., 2019; Liu et al., 2020). At present, the most commonly used material for prostheses on the market is titanium. Therefore, many studies have focused on surface modification of titanium to enhance its osteoconduction and osteointegration (Liu et al., 2020). Among them, nanotubes fabricated on the surface of titanium by anodic oxidation etching have been reported in the literature, which can significantly enhance the ability of bone marrow-derived mesenchymal stem cells to differentiate into osteoblasts (Wang et al., 2014; Zhang et al., 2016). Nanotubes can significantly enhance the adhesion and proliferation of mesenchymal stem cells (Zhang et al., 2016). At the same time, different tube diameters and lengths will exert different effects on the osteogenic differentiation ability of mesenchymal stem cells (Park et al., 2007; Wang et al., 2011). However, how stem cells perceive the difference in titanium surface topography, transform it into internal biological signals, and finally promote osteogenesis remains to be further studied.

Bone marrow-derived mesenchymal stem cells are a kind of stem cell with multidirectional differentiation ability (Chang et al., 2019; Park et al., 2019; Yong et al., 2020). Under different induction and stimulation conditions, they will differentiate into three lines: osteogenic, chondrogenic, and adipogenic cells. Studies have shown that different mechanical stimuli and different substrate stiffness will affect the differentiation of mesenchymal stem cells in different directions (Curran et al., 2006; Sun et al., 2012; Perestrelo et al., 2018). Because the stiffness of bone tissue is higher, such an environment will be more conducive to the differentiation of mesenchymal stem cells in the direction of osteogenesis. The common point of mechanical stimulation and substrate stiffness in controlling stem cell differentiation is to change the topography of cells and rearrange the cytoskeleton (Chang et al., 2019). Previous studies have proved that the uneven topography of nanotubes changes the topography of mesenchymal stem cells and promotes osteogenesis through the rearrangement of F-actin (Tong et al., 2020). However, how the physical stimulation of nanotopography changes the internal signal pathway and the final effector molecule are still unknown.

Yap is an important component of the Hippo signal pathway. When the Hippo pathway is turned on, a series of kinases upstream of Yap, such as Mst1/2 and Lats1/2, are activated to further phosphorylate Yap and Taz to prevent them from entering the nucleus and starts the regulation of downstream transcription factors. When the pathway is closed, Yap/Taz will be dephosphorylated and activated and then enters the nucleus to activate the downstream transcription factor TEAD family and start the transcription of downstream genes such as Ctgf and Cyr61 (Dupont et al., 2011; Lorthongpanich et al., 2019). The Hippo pathway has been widely proved to be involved in the process of tumor proliferation and metastasis (Fan et al., 2021; Hasegawa et al., 2021), and Yap has also been proved to be a mechanosensor, which plays an important role in osteogenesis promoted by mechanics (Dupont et al., 2011; Du et al., 2019). In addition to osteogenesis, the Hippo pathway is also involved in the differentiation and degeneration of cartilage (Deng et al., 2018) and osteoclasts (Yang et al., 2021). It plays an important role in maintaining bone homeostasis and promoting bone integration. However, what role it plays in the process of titanium nanotopography promoting osteogenesis and its mechanism are still controversial.

Piezo is a family of newly discovered mechanosensitive cation channels on the surface of the cell membrane in recent years (Sugimoto et al., 2017; Wu et al., 2017). It is divided into two subtypes: Piezo1 and Piezo2. Piezo2 is mainly distributed in sensory tissues, such as dorsal root ganglion sensory neurons and Merkel cells. Piezo1 is widely distributed in non-sensory tissues such as chondrocytes, the bladder, endothelial cells, the kidney, and red blood cells (Wu et al., 2017). Recent literature studies generally suggest that Piezo1 may be involved in the maintenance of bone homeostasis (Hao et al., 2019; Li et al., 2019; Wang et al., 2020). Asuna et al. (Sugimoto et al., 2017) found that the expression of Piezo1 increased in the process of mechanical pressure promoting osteogenesis, and the use of Yoda1, an agonist of Piezo1, can significantly promote the differentiation of stem cells into osteogenesis. Conditional knockout of the *Piezo1* gene in osteoblasts can significantly reduce bone mass and bone formation in mice. Mice with conditional Piezo1 knockout in osteoblasts and their progeny cells showed higher risks of spontaneous fracture and significantly enhanced bone resorption (Wang et al., 2020). However, the role of Piezo1 in nanotopography promoting osteogenesis and the reason behind its expression regulation and function have not been clearly determined. Hasegawa et al. found that the transcriptional activity of Piezo1 was regulated by the Yap-TEAD compound in oral squamous cell carcinoma cells and whether such regulation plays a role in osteogenesis remains to be explored (Hasegawa et al., 2021).

In this study, we found that the nanotopography of the titanium surface can significantly promote the osteogenic differentiation of bone marrow-derived mesenchymal stem cells *in vivo* and *in vitro*. By adoption of the Yap inhibitor verteporfin and knockdown and overexpression of Yap, it is proved that Yap is involved in the process of mesenchymal stem cells sensing the changes of physical topography and transforming them into biological signals. It is proposed and preliminarily proved that Piezo1, which has been widely studied in the orthopedic field in recent years, is a downstream key molecule for nanotopography to promote osteogenesis through Yap.

## Materials and Methods

### Fabrication of TiO_2_ Nanotubes

Our method of fabricating nanotubes on titanium surfaces has been explained in detail in our previous work (Tong et al., 2020). In brief, pure titanium slices were polished and then washed sequentially with anhydrous alcohol and deionized water in an ultrasonic cleaning machine. Titanium was fixed as the anode and a platinum piece as the cathode in a solution of 0.15 M NH_4_F and 90% glycol for 1 h under 50 V.

### Surface Characterization

Samples fabricated at 50 V were washed with deionized water and dried at room temperature. Scanning electron microscopy (SEM450, FEI Nova Nano SEM; Thermo Fisher Scientific, Waltham, MA, United States) was employed to scan and analyze the structure of nanotubes, including their inner diameters. In addition, X-ray energy dispersive analysis (EDS, Xplore30, Oxford Instrument, Oxford, United Kingdom) was performed to analyze the elemental composition of nanotubes.

### Cell Lines and Reagents

The osteoblast-like cell line MC3T3-E1 was purchased from the Cell Bank of the Chinese Academy of Sciences (Shanghai, China) and was cultured in α-minimal essential medium (*α*-MEM; Gibco, Thermo Fisher Scientific, Waltham, MA, United States) with 10% fetal bovine serum (FBS) and 1% penicillin-streptomycin (Gibco, Thermo Fisher Scientific, Waltham, MA, United States) solution. Verteporfin was purchased from MCE (New Jersey, United States) and dissolved in DMSO. To avoid the cytotoxicity of DMSO, the final concentration of DMSO in the culture medium was less than 0.1%.

### BMSC’s Isolation and Cell Culture

Four-week-old male Sprague–Dawley (SD) rats were purchased from the experimental animal center of Shanghai Ninth People’s Hospital (Shanghai, China). Rat femurs and tibias were separated aseptically, and bone marrow-derived mesenchymal stem cells (BMSCs) were collected by rinsing the medullary cavity with culture medium. BMSCs were further cultured in α-minimal essential medium with 10% fetal bovine serum (FBS) and 1% penicillin-streptomycin (Gibco, Thermo Fisher Scientific, Waltham, MA, United States) solution. BMSCs were incubated at 37°C in a humidified atmosphere consisting of 95% air and 5% CO_2_. Only cells between passages three and seven were used for experiments. Osteogenic induction medium (Cyagen, United States) was composed of growth medium supplemented with 100 nM dexamethasone, 10 mM *ß*-glycerophosphate, and 50 μM ascorbic acid.

### Cell Proliferation

A total of 24-well-size smooth titanium slices and titanium slices for nanotopography were placed in a 24-well cell culture plate. BMSCs were cultured at a density of 3×10^4^ cells per well. Cell viability and proliferation were assessed using Cell Counting Kit-8 (CCK8) (Dojindo, Kumamoto, Japan) at 24, 48, and 72 h after seeding. At the end of each experimental period, cells were incubated with 50 μl of the CCK-8 reagent and 500 μl *α*-MEM for 2 h at 37°C. Optical density values were recorded on a spectrophotometer at 450 nm on an Infinite M200 PRO multimode microplate reader (Tecan Life Sciences, Männedorf, Switzerland).

### Construction of Stable Knockdown/Overexpression Cell Line

Knockdown/overexpression lentiviruses of Yap labeled with GFP and puromycin resistance were purchased from OBIO, Shanghai. First, MC3T3-E1 cells were seeded on a six-well plate at a density of 1.5×10^5^ per well. After cells adhered to the wall, a corresponding amount of virus was added to the culture medium according to an optimal multiplicity of infection (MOI) of 20. To promote the entry of virus into the cells, polybrene was added in a ratio of 1:200. After 48 h of infection, the fluorescence intensity was observed under the fluorescence microscope to help determine the infection efficiency. Puromycin was added to screen the resistant cells. After 24 h, the fresh medium was replaced to obtain a stable Yap knockdown/overexpression MC3T3-E1 cell line.

### Alkaline Phosphatase Staining and ALP Activity Analysis

BMSCs and MC3T3-E1 cells were seeded onto smooth titanium slices, nanotube titanium slices, or 24-well plates at a density of 3×10^4^ per well and cultured with osteogenic medium. After 7 days of incubation, cells were washed three times with PBS for 5 minutes each, fixed with 4% paraformaldehyde for 10 minutes, and washed with PBS three times again. Finally, cells were incubated in the BCIP/NBT alkaline phosphatase color development kit, according to the manufacturer’s instructions (Beyotime Institute of Biotechnology, Jiangsu, China).

For alp activity analysis, cells were first lysed with RIPA buffer without protease and phosphatase inhibitors, and then, the centrifuged lysates were assayed using an ALP Assay Kit (Beyotime Institute of Biotechnology, Jiangsu, China) following the protocol provided.

### Alizarin Red S Staining

Cells were cultured in 24-well plates at a density of 1×10^5^ cells per well and cultured in osteogenic medium for at least 21 days. During the late period of osteogenic differentiation, calcium deposition in the osteoblast was examined by alizarin red S staining. In brief, we used PBS to clean the cultured cells three times, fixed the cells for 10 min in 4% PFA, and stained them with 1% alizarin red S solution (Cyagen, United States) for 10 min. To remove non-specific staining, we used 50% ethanol to clean the stained cells thoroughly. Calcium deposits were represented by positive red staining, and representative images were taken.

### Immunocytochemistry

For immunofluorescence, cells were seeded on smooth and nano-topographic titanium slices at a density of 1 × 10^5^ cells per well for two days. After washing with PBS, 4% PFA was used to fix the BMSCs for 10 min, and 0.3% Triton X-100 was used to permeabilize the cell membrane for 5 min after washing three times with PBS. After blocking with 10% goat serum (Solarbio, Beijing, China) for 1h, the cells were incubated with a primary antibody for YAP (diluted 1:200, purchased from Proteintech, United States) overnight at 4°C. After 1 day, the cells were washed three times with PBS for 5 mins. Then, they were incubated with an Alexa Fluor 555 conjugate secondary antibody (anti-rabbit, 1:200; Abcam, United Kingdom) for 2 h and subsequently incubated with DAPI (Beyotime Institute of Biotechnology, Jiangsu, China) for 5 min. Finally, after PBS wash again, fluorescence images were captured with a confocal microscope (Olympus, Inc., Tokyo, Japan).

### Protein Extraction and Western Blotting

After appropriate treatment, cells were rinsed with PBS and lysed with RIPA (Beyotime Institute of Biotechnology, Jiangsu, China) added with protease and phosphatase inhibitors (Thermo Fisher Scientific, Waltham, MA, United States) for 10 min on ice. Protein was collected as the supernatant after a centrifugation of 12,000×*g* for 15 min. Isolation of nuclear and cytoplasmic proteins was achieved with a kit from Beyotime (P0028). Protein concentration was measured by using a BCA kit, and total protein underwent denaturation after being boiled at 99°C for 10 min with a loading buffer. An equal amount of protein was loaded onto a 4–20% SDS- PAGE gel, and proteins with different molecular weights were separated by electrophoresis and then electroblotted onto a PVDF membrane. TBST with 5% bovine serum albumin was used to block unspecific antigen on the membrane for 1 h at RT, and the membrane was incubated with the primary antibody at 4°C overnight. After being washed with TBST, the membrane was incubated with the secondary antibody for 2 h at RT, and protein immunoreactivity was detected on a LI-COR Odyssey fluorescence imaging system (Odyssey, LI-COR Biosciences, Lincoln, NE, United States). Primary antibodies used in this study included Piezo1 (15939-1-AP, Proteintech), *ß*-actin (ab8227, Abcam), Runx2 (12556S, Cell Signaling Technology), osteopontin (ab8448, Cell Signaling Technology), and anti-Yap (13584-1-AP, Proteintech). Secondary antibodies were anti-rabbit IgG (H + L; DyLight™ 800 4× PEG conjugate; Abcam).

### Quantitative Real-Time PCR

Quantitative Real-time PCR was performed after cells were treated for the appropriate number of days, according to the conditions required for each experiment. Total RNA was extracted from cells using a total RNA Kit (R6812-01HP, Omega Bio-Tek Inc., Norcross, GA, United States). The concentration of RNA samples was determined by optical density at 260 nm wavelength. RNA samples were reverse-transcribed into cDNA with a cDNA synthesis kit (Takara, Shiga, Japan), according to the manufacturer’s instructions. Quantitative Real-time PCR was performed with QuantStudio six Flex real-time PCR system (Life Technologies) with a SYBR Green kit (Bimake) for quantitative real-time PCR of Ctgf, Cyr61, Piezo1, Runx2, Opn, Osx, Ocn, Col1a1 and Alpl. The final volume is 10μl, including 1 μl cDNA, 5 μl SYBR Green Mix, 0.4 μl upstream and 0.4 μl downstream primers, and 3.2 μl dH2O. *Gapdh* was used as an internal reference, and the expressions of different genes were analyzed with the 2-ΔΔCt method and expressed as fold changes compared to the expression of *Gapdh*. The experiment was repeated three times independently. Primers for each gene were designed by Primer Premier 6.0 software. The sequences of the primers used are listed in Table 1.

**TABLE 1:** Primers used in the qRT-PCR assay.

Gene	Organism	Forward (5–3′)	Reverse (5–3′)
*Gapdh*	*Rattus norvegicus*	GGC​AAG​TTC​AAC​GGC​ACA​G	CGC​CAG​TAG​ACT​CCA​CGA​CAT
*Col1a1*	*Rattus norvegicus*	TGA​TGG​ACC​TGC​TGG​CTC​TC	GAC​CAC​GTT​CAC​CAC​TTG​CT
*Osx*	*Rattus norvegicus*	CCA​ATG​ACT​ACC​CAC​CCT​TTC​C	ATGGATGCCCGCCTTGTA
*Ocn*	*Rattus norvegicus*	GGA​CCC​TCT​CTC​TGC​TCA​CTC​TG	ACC​TTA​CTG​CCC​TCC​TGC​TTG​G
*Opn*	*Rattus norvegicus*	TGA​TGA​CGA​CGA​CGA​TGA​CGA​C	TGT​GCT​GGC​AGT​GAA​GGA​CTC
*Alpl*	*Rattus norvegicus*	GAC​AAT​GAG​ATG​CCG​CCA​GAG	CAT​CCA​GTT​CAT​ATT​CCA​CAT​CAG​TTC
*Gapdh*	*Mus musculus*	GGC​AAG​TTC​AAC​GGC​ACA​G	CGC​CAG​TAG​ACT​CCA​CGA​CAT
*Runx2*	*Mus musculus*	AGA​CCA​GCA​GCA​CTC​CAT​ATC​TCT	CGT​CAG​CGT​CAA​CAC​CAT​CAT​TCT
*Osx*	*Mus musculus*	AAG​TTC​ACC​TGC​CTG​CTC​TGT​TC	GGC​GGC​TGA​TTG​GCT​TCT​TCT​T
*Ocn*	*Mus musculus*	AAG​CAG​GAG​GGC​AAT​AAG​GTA​GTG	TCT​TCA​AGC​CAT​ACT​GGT​CTG​ATA​GC
*Opn*	*Mus musculus*	GAC​GAT​GAT​GAT​GAC​GAT​GGA​GAC​C	CTG​TAG​GGA​CGA​TTG​GAG​TGA​AAG​TG
*Alpl*	*Mus musculus*	TCA​CGG​CGT​CCA​TGA​GCA​GAA	TAC​AGG​CAA​GGC​AGA​TAG​CGA​ACT
*Ctgf*	*Mus musculus*	ACA​CCG​CAC​AGA​ACC​ACC​ACT​C	TAA​TGG​CAG​GCA​CAG​GTC​TTG​ATG​AAC
*Cyr61*	*Mus musculus*	ATA​CTG​CGG​CTC​CTG​CGT​AG	CCT​GAA​CTT​GTG​GAT​GTC​ATT​GAA​TAG
*Axl*	*Mus musculus*	CTT​GTG​TCC​ATT​CAA​CTG​TGC​TAC​G	TTC​CAT​CCT​CTT​GCC​GCT​CAG
*Yap*	*Mus musculus*	GCC​TAC​ACT​GGA​GCA​GGA​TGG​A	GAT​AGG​TGC​CAC​TGT​TAA​GAA​AGG​GAT
*Piezo1*	*Mus musculus*	AGT​ATC​TGC​TTC​TTC​TTC​CTG​CTC​TTG	GAC​TTC​TCC​TCA​ATC​TGG​CGA​TGG

### Surgical Procedure

Animal experiments and surgery were conducted in the animal laboratory of Shanghai Ninth People’s Hospital affiliated with Shanghai Jiaotong University. National Laboratory Animal Care and Use guidelines were followed. The animal experiment plan was approved by the ethics committee of Shanghai Ninth People’s Hospital. The preparation of nanotopography titanium nails is the same as the method described before. Twelve 6-week-old male SD rats were randomly divided into two groups: 1) the smooth titanium nail group and 2) nanotopography titanium nail group. During the operation, the rats were anesthetized by an intraperitoneal injection of 4% chloral hydrate; the skin was prepared and disinfected with iodophor. After cutting the knee joint skin, the distal femur was exposed and a hole with a diameter of 2 mm and a depth of 4 mm was drilled at the lateral epicondyle of the femur from medial to lateral with a surgical drill. A sterilized titanium rod was implanted into the hole. After implantation, the muscle tissue and skin were sutured at different levels. The rats were sacrificed 2 months after the operation, and the femurs were collected and fixed in 4% PFA for subsequent analysis.

### Micro-CT and Histological Evaluation

We used a high-resolution Micro-CT scanner (skyscan 1072; Skyscan, Aartselaar, Belgium) to scan the amount of new bone around the implant. 0.5 μm thickness of bone around the implant was defined as the new bone and analyzed. We measured the microstructure index of trabecular bone mineral density (BV/TV), trabecular thickness (TB. Th), trabecular number (TB. N), and trabecular spacing (TB. SP). For histological evaluation, pre-fixed femoral specimens were decalcified in 10% ethylenediaminetetraacetic acid (EDTA). After decalcification, the implant was gently removed and embedded into paraffin to perform tissue section (5 μm). For histological evaluation, tissue sections were stained with hematoxylin and eosin (H&E). To evaluate immunohistochemistry, tissue sections were dewaxed in xylene and standard alcohol gradients and then washed with PBS. Then, nonspecific binding sites were blocked with 10% goat serum. The sections were incubated with primary antibodies (anti-OCN, anti-Piezo1, and anti-Yap purchased from Proteintech) at 4°C overnight. The next day, sections were washed with PBS and incubated with the appropriate HRP-labeled secondary antibody (Abcam) at RT for 1 h and then further developed with diaminobenzidine solution.

### Statistical Analysis

SPSS 18.0 software was used for all statistical analysis. All data are representative of at least three independent experiments unless otherwise indicated. Data are expressed in the form of mean ± standard deviation. Differences between three or more groups were evaluated by one-way analysis of variance followed by the Student–Newman–Keuls *post hoc* test, and differences between two groups were analyzed by Student’s t test. Graphpad 8.0 was used to draw all relevant figures. *p* values < 0.05 were considered statistically significant. All quantitative data were distributed normally.

## Results

### Nanotubes Fabricated by Anodic Oxidation Show Satisfactory Lumen Structure and Biocompatibility

**FIGURE 1:**
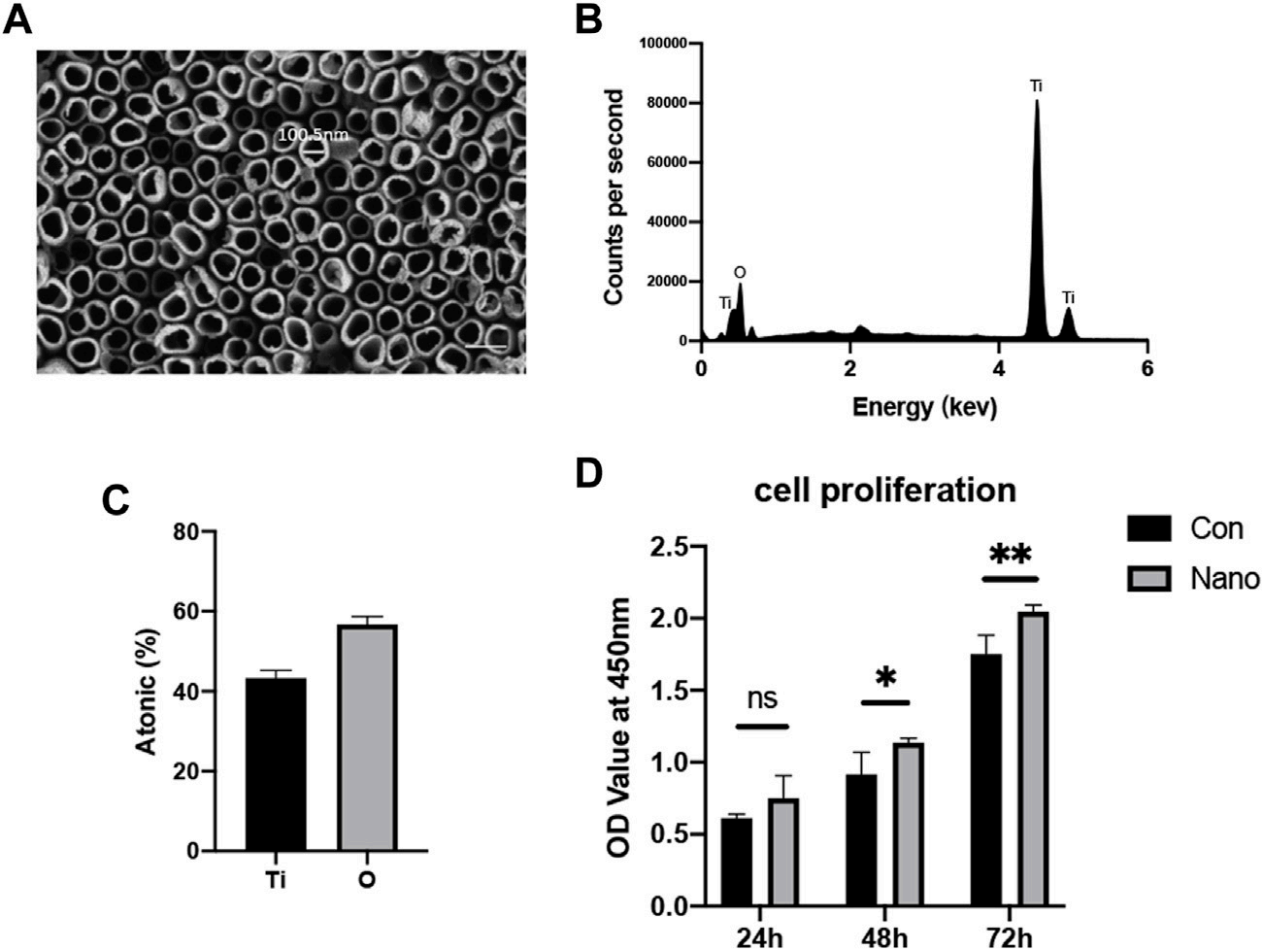
Nanotopography surface characteristics and biocompatibility. **(A)** Lumen structure of nanotubes observed by SEM with a diameter of approximately 100 nm. Scale bar: 200 nm. **(B)** EDS analysis of the chemical element composition of nanotubes. **(C)** Ti and O element ratio on nanotopography. **(D)** CCK8 test of BMSCs seeded on nanotubes and smooth titanium. *p < 0.05, **p < 0.01.

SEM results show that nanotubes fabricated at 50 V constant pressures are evenly distributed on the surface of titanium. The inner diameter of a nanotube is approximately 100 nm, and there are no residual impurities on the nanotube surface after ultrasonic vibration washing (Figure 1A). The results of energy spectrum scanning also show that titanium and oxygen account for the highest proportion in the element composition, and the element proportion is about 1:2, indicating that the main component of nanotubes is titanium dioxide (Figures 1B,C). The CCK8 results of 24, 48, and 72 h showed that compared with a smooth surface, titanium with nanotopography had no adverse effect on the proliferation of mesenchymal stem cells in short-term or long-term culture (Figure 1D). The aforementioned results show that nanotubes have satisfactory biocompatibility and will not produce obvious toxicity to cells.

### Nanotopography Can Promote Osteogenesis *In Vivo* and *In Vitro*

**FIGURE 2:**
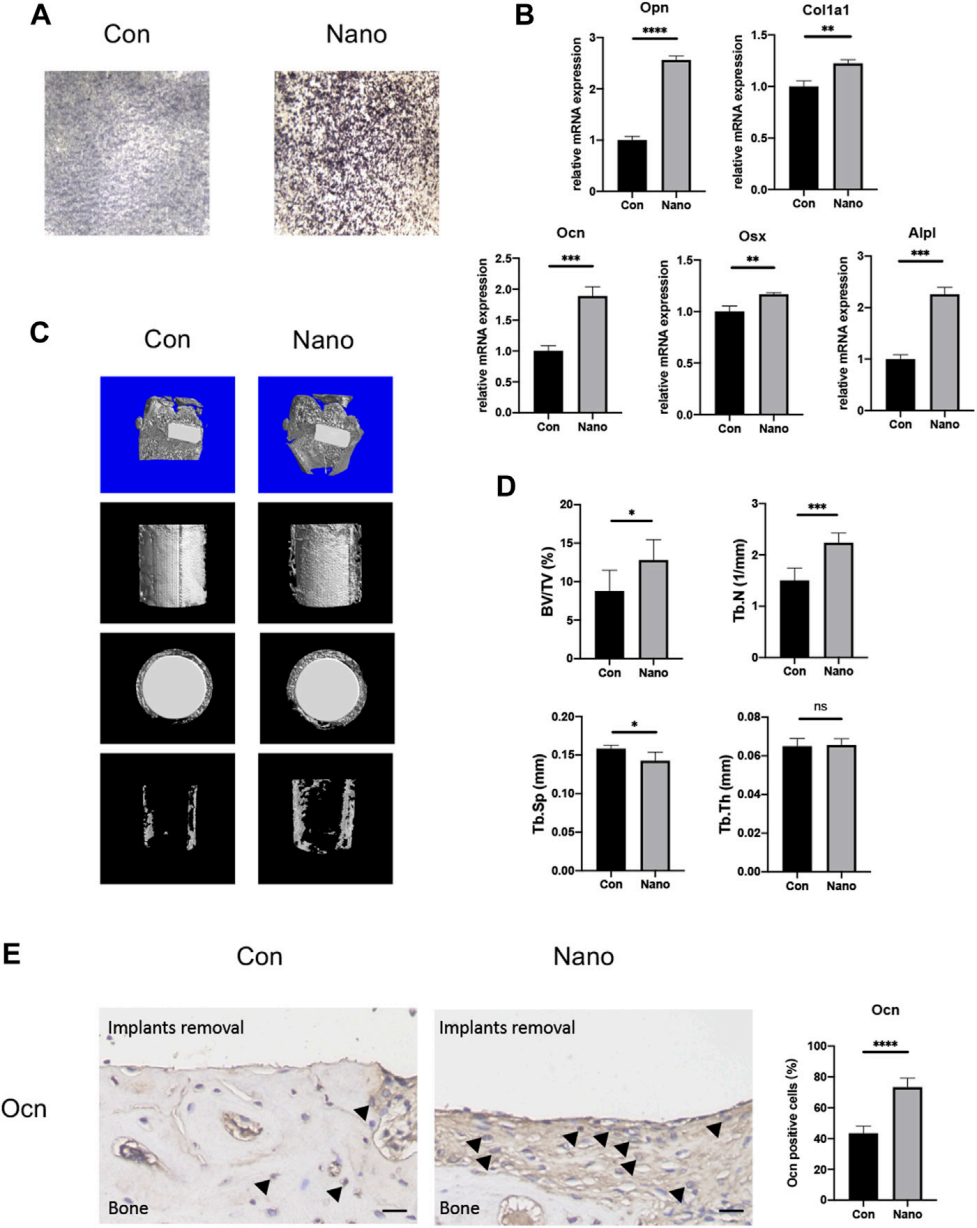
Nanotopography promotes osteogenic differentiation of BMSCs both *in vivo* and *in vitro*. **(A)** ALP staining of BMSCs on different topographies. **(B)** Gene expression of osteogenic differentiation markers of BMSCs seeded on various topographies. **(C)** Micro-CT scans of the distal femur and presentative reconstruction of the new bone around implants from different views. **(D)** Analysis of BV/TV, Tb. N, Tb. Sp, and Tb. Th for new bones around implants with different topographies. **(E)** Immunohistochemistry staining and quantitative analysis of Ocn in new bones around implants. Scale bar: 10 μm *p < 0.05, **p < 0.01, ***p < 0.001, ****p < 0.0001.

It is reported that nanotopography with different diameters will affect the osteogenic ability of BMSCs. Our results showed that nanotubes with an inner diameter of 100 nm could significantly increase the mRNA expression of osteogenic genes such as *Col1a1*, *Alpl*, *Runx2*, *Ocn*, *Opn*, and *Osx in vitro*. ALP staining also showed that nanotubes could significantly increase the expression of alkaline phosphatase, which is one of the markers of osteogenesis (Figures 2A,B). At the same time, we implanted titanium rods with different topographies in the distal femur condyles of rats to verify the osteogenic effect of nanotopography *in vivo*. The results of micro-CT showed that the BV/TV value of new bone trabeculae around the implant in the nanotopography group was higher. The number of bone trabeculae was significantly greater than that in the control group (Figures 2C, D). After removing the titanium rod, immunohistochemistry of Ocn also showed that the new bone around the implant in the nano topography group was significantly more than that in the control group (Figure 2E). Our results show that nano topography can significantly promote differentiation and osteogenesis of BMSCs *in vivo* and *in vitro*.

### Yap is Involved in Nano Topography Osteogenesis

**FIGURE 3:**
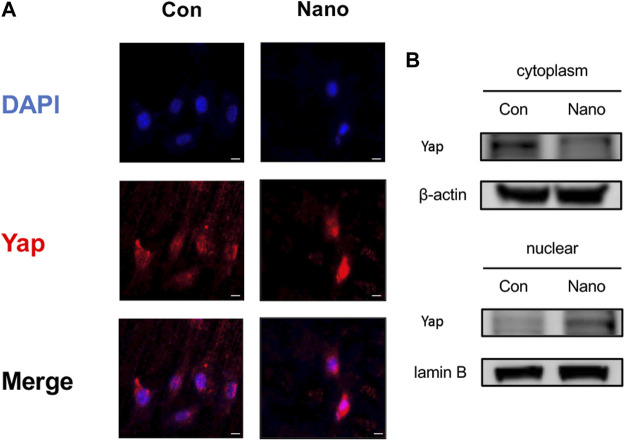
Yap entering the nucleus plays an important role in nanotopography-related osteogenesis. **(A)** Immunofluorescence staining of Yap proved the accumulation of Yap in the nucleus under stimulation of nanotubes. Scale bar: 10 μm. **(B)** Western Blot of Yap in the nucleus and cytoplasm proved its accumulation in the nucleus when seeded on nanotubes.

BMSCs were seeded on smooth and nanotopography titanium slices, respectively, for cell immunofluorescence staining of Yap. It was found that the distribution of Yap was more concentrated in the nucleus in the nano topography group (Figure 3A). Yap activation promotes the expression of downstream genes by entering the nucleus. Therefore, the results show that Yap activation in the nucleus may be involved in the process of nanotopography promoting osteogenesis. In addition, when we separated the cytoplasmic and nuclear proteins, we found that the distribution of Yap in the nucleus was significantly higher in the nanotopography group than that in the control group (Figure 3B).

### Inhibition of Yap or Knockdown/Overexpression of Yap Can Affect the Osteogenesis of MC3T3-E1 Cells

**FIGURE 4:**
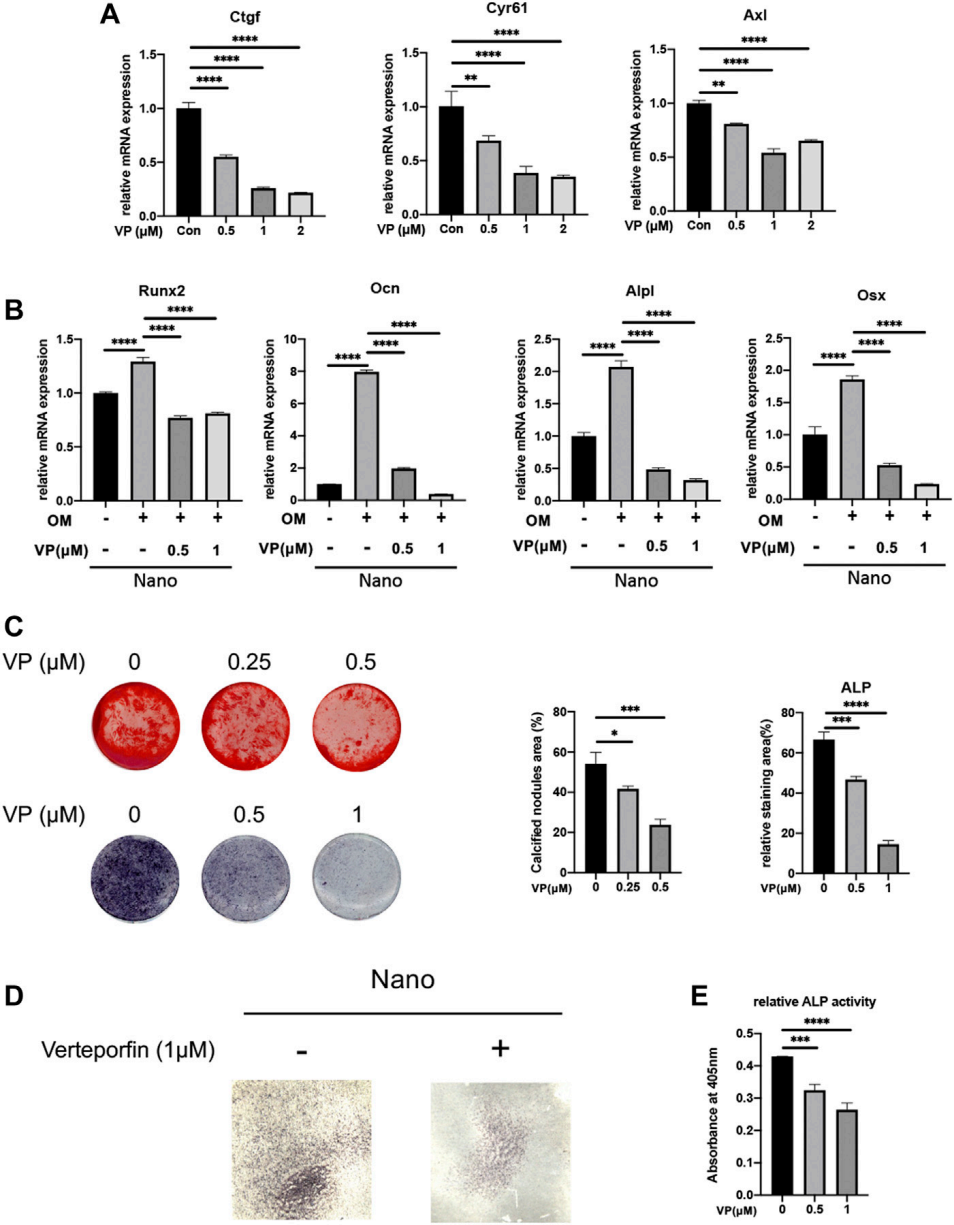
Yap inhibitor, verteporfin, inhibits osteogenic differentiation of MC3T3-E1 cells seeded on nanotubes or 24-well plates. **(A)** Gene expression of Yap downstream genes Ctgf, Cyr61, and Axl under different concentrations of verteporfin. **(B)** Expression of osteogenic genes of MC3T3-E1 cells seeded on nanotubes. OM: osteogenic induction medium. **(C)** Alizarin Red S staining and ALP staining of MC3T3-E1 cells seeded on 24-well plates and quantitative analysis of calcified nodules areas and relative ALP staining areas. **(D)** ALP staining of MC3T3-E1 cells seeded on nanotubes with verteporfin. **(E)** Relative ALP activities of MC3T3-E1 cells on nanotubes treated with verteporfin. *p < 0.05, **p < 0.01, ***p < 0.001, and ****p < 0.0001.

**FIGURE 5:**
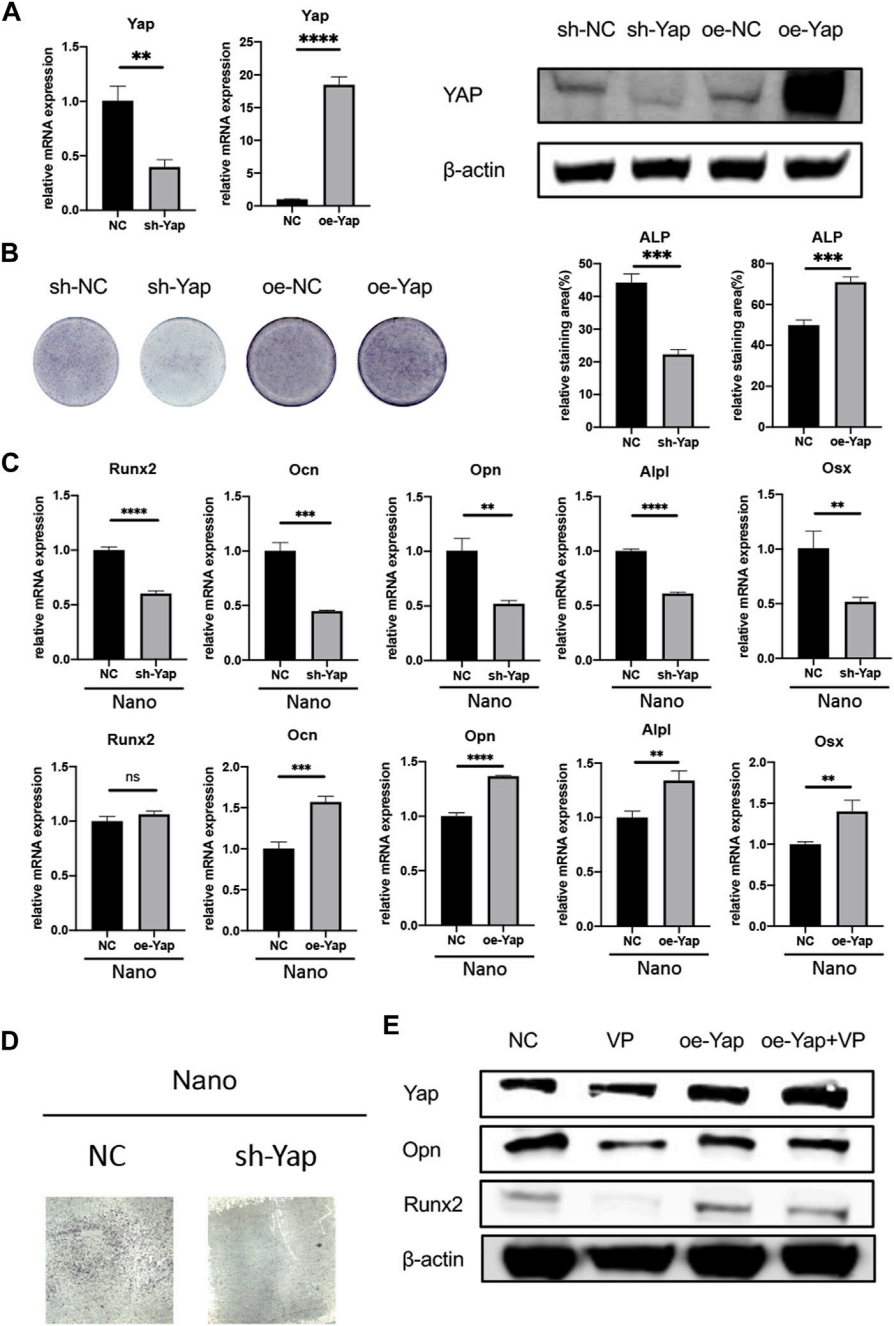
Knockdown/overexpression of Yap regulates osteogenesis. **(A)** Verification of knockdown and overexpression of Yap through rt-qPCR and Western Blot. oe, overexpression. **(B)** ALP staining of sh-Yap and oe-Yap and quantitative analysis of the relative staining area. **(C)** Expression of osteogenic genes of sh-Yap and oe-Yap on nanotopography. **(D)** ALP staining of sh-Yap cells on nanotubes. **(E)** Overexpression of Yap could rescue anti-osteogenic effect of verteporfin. *p < 0.05, **p < 0.01, ***p < 0.001, and ****p < 0.0001.

To stably manipulate the expression of Yap, we chose the MC3T3-E1 cell line for further research. In order to verify the effect of Yap on osteogenesis and its role in nano topography osteogenesis, we selected verteporfin, an inhibitor of Yap, for experiments. First, when VP with different concentration gradients treated cells, the expression of Yap downstream genes such as Ctgf and Cyr61 were significantly inhibited (Figure 4A). When BMSCs seeded on titanium nanotubes were treated with 0.5 and 1 μM verteporfin for 7 days, it was found that the expression of osteogenic genes such as *Alpl*, *Runx2*, *Ocn*, *Opn*, and *Osx* decreased significantly (Figure 4B), and the results of ALP staining and AR staining of osteogenesis showed that verteporfin could significantly inhibit osteogenesis whether MC3T3-E1 cells were seeded on titanium or not (Figures 4C,E). Then, we constructed a Yap knockdown/overexpression cell line to further explain the role of Yap in osteogenesis (Figure 5A). The results of real-time quantitative PCR and ALP staining showed that the osteogenesis on nanotubes was inhibited when Yap was knockdown, while osteogenesis was significantly enhanced when Yap was overexpressed (Figures 5B–D). Finally, in order to further confirm the conclusion of this research, we carried out the rescue experiment. Western blot results showed that the overexpression of Yap could significantly rescue the inhibitory effect of VP on osteogenesis of BMSCs on nanotubes (Figure 5E). Results show that Yap is involved in the process of osteogenesis and plays an important role in the promotion of osteogenesis by nano topography.

### Yap Can Regulate the Expression of Piezo1

**FIGURE 6:**
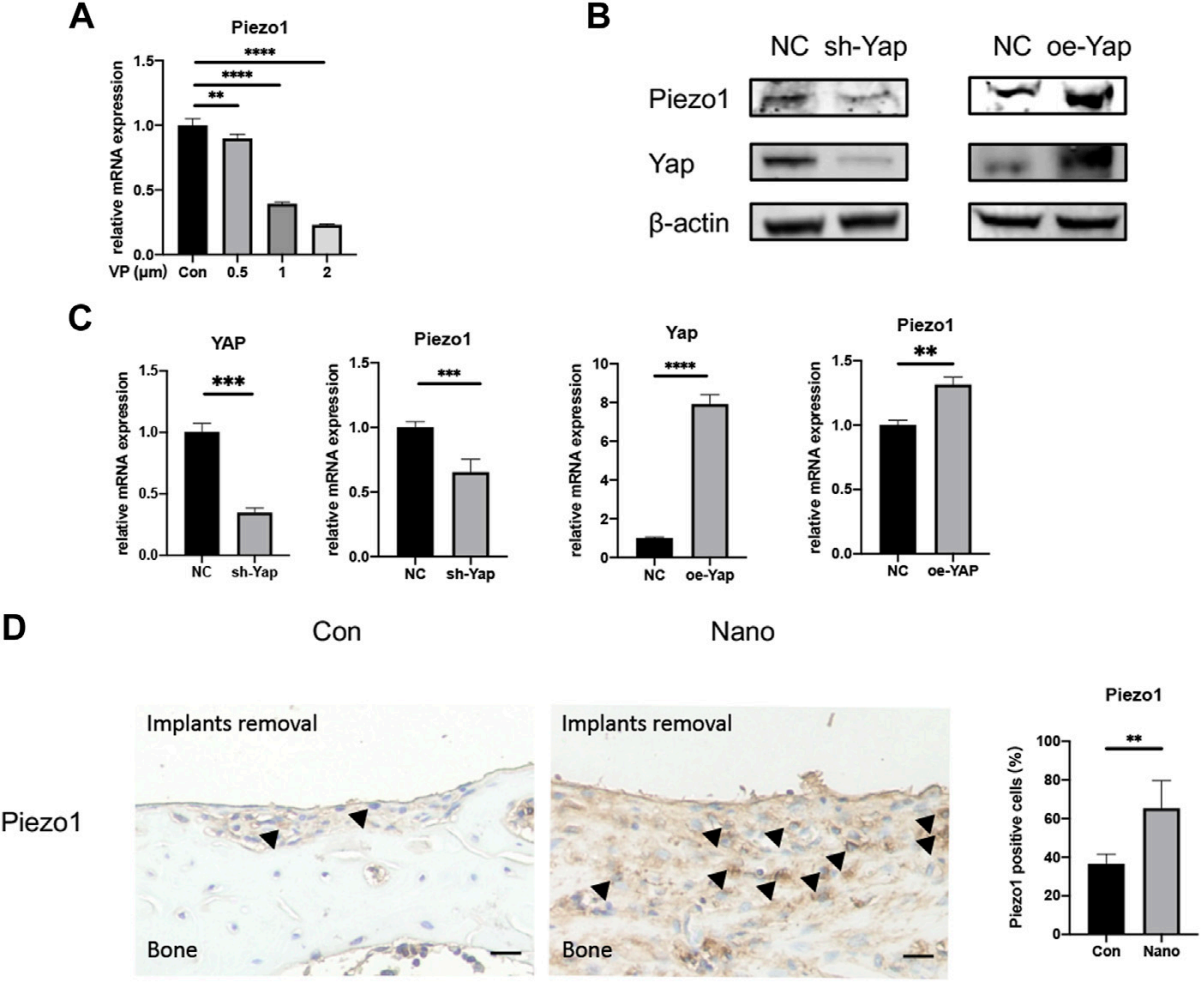
Yap could regulate the expression of Piezo1. **(A)** Verteporfin inhibits expression of Piezo1 in a dose-dependent manner. **(B)** Knockdown or overexpression of Yap could regulate the protein expression of Piezo1. oe, overexpression. **(C)** Knockdown or overexpression of Yap could regulate the mRNA expression of Piezo1. **(D)** Expression of Piezo1 was elevated in bones around defects by immunohistochemistry and quantitative analysis. Scale bar: 10 μm *p < 0.05, **p < 0.01, ***p < 0.001, and ****p < 0.0001.

**FIGURE 7:**
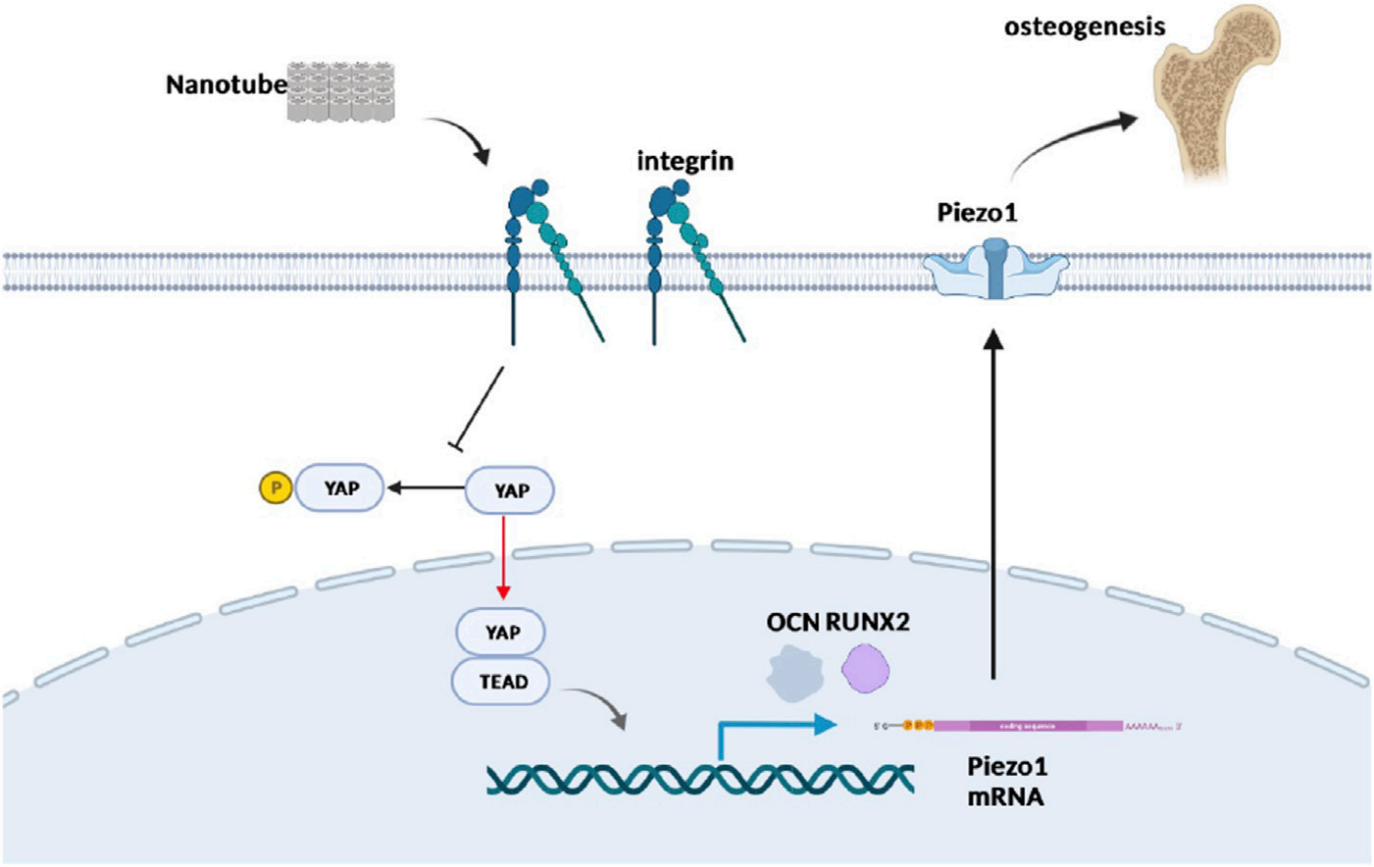
Schematic plot to illustrate the role of Yap and Piezo1 in promoting nanotube-related osteogenesis. Nanotopography is recognized by mechanosensors such as integrins residing in the cell membrane, which suppresses the Hippo pathway and promotes nuclear entry of Yap. Furthermore, Yap activates TEADs and enhances the expression of Piezo1, which has been proved to be significant in osteogenesis. Created in BioRender.com.

It has been reported that Yap may regulate the expression and function of Piezo1 in tumor cells to regulate its proliferation and migration (Hasegawa et al., 2021). This study also preliminarily explored the possible downstream mechanism of Yap regulating nanotopography osteogenesis by regulating the expression of the Piezo1 gene. First, we used the Yap inhibitor VP with different concentration gradients to treat cells. It was found that the mRNA expression of Piezo1 decreased significantly with the increase of VP concentration (Figure 6A). Then, we detected the mRNA and protein expression of Piezo1 in Yap knockdown/overexpression cell lines constructed as earlier. The results of real-time quantitative PCR and Western blot showed that the expression of Piezo1 decreased when Yap was knocked down, while the expression of Piezo1 increased when Yap was overexpressed (Figures 6B,C). This suggests that Piezo1 may be one of the downstream mechanisms of Yap regulating nanotopography osteogenesis. In addition, in sections of immunohistochemistry, the expression of Piezo1 in newborn bones in the nanotopography group is evidently higher than that in the control group (Figure 6D). A diagram of this research is illustrated in Figure 7.

## Discussion

This study confirmed that fabrication of nanotubes on titanium surfaces by anodic oxidation can promote the osteogenic differentiation of mesenchymal stem cells both *in vivo* and *in vitro* and verified that Yap plays an important role in this process. In addition, we also verified the possible downstream regulatory gene *Piezo1* of Yap regulating osteogenesis, which provides an effective intervention target for promoting bone integration between orthopaedic plants and bone tissue *in vivo* in the future.

In the process of fracture healing and implant osteointegration, the most critical step is the adhesion and aggregation of bone marrow-derived mesenchymal stem cells locally and their differentiation into osteoblasts (Shah et al., 2019). After nanotubes are fabricated on a titanium surface, there will be a gap with a diameter of about 100 nm between the nanotubes and the center of the nanotubes, which can accommodate the synapses of cells so as to promote the adhesion and proliferation of stem cells (Reznikov et al., 2018). Our CCK8 data also showed that a titanium sheet with nano topography could significantly enhance the proliferation of cells compared with the control group. In terms of differentiation, many literature studies have reported that both mechanical signals such as tensile force and physical signals such as material surface topography and stiffness can promote BMSC’s osteogenesis once appropriate stimulation frequency and time are applied (Arnsdorf et al., 2009; Shi et al., 2011; Shi et al., 2012). Arnsdorf et al. (Arnsdorf et al., 2009) reported that 1 hour of oscillatory fluid flow with a peak shear stress of 1.0 Pa evidently increases the expression of Runx2. In contrast, Shi et al.(Shi et al., 2012) found that 3% continuous cyclic mechanical tension inhibited osteogenic differentiation of MSCs, which emphasized the importance of stimulation frequency. Data in this study also prove that nanotubes fabricated at a 50 V constant pressure can promote the expression of osteogenic genes and the formation of new bone both *in vivo* and *in vitro*. The mechanism of this kind of physical signal promoting osteogenesis has also been widely discussed. At present, the recognized mechanism is that the physical signal of external force and material stimulates the mechanical receptors on the cell surface by changing the morphology of the cell and then further changing the F-actin cytoskeleton in the cell so as to activate the downstream pathway (Arnsdorf et al., 2009; Tong et al., 2020). However, it is still unclear how mechanical signals activate the downstream signal pathway in osteogenesis.

At present, there are two kinds of mechanical receptors widely studied. One is integrin, which mediates the interaction between cells and the extracellular matrix and is composed of two subunits, *α* and *ß*. Subunit *α* has 18 subtypes while *ß* has eight subtypes; the main integrin type in mesenchymal stem cells is *α*5*β*1. It has been reported in the literature that *α*5 (Cha et al., 2020; Zhang et al., 2021), *α*V (Lopes et al., 2019), *α*11 (Shen et al., 2019), and other subunits are involved in the process of osteogenic differentiation. Lopes et al. (Lopes et al., 2019) knocked down integrin *α*V in MC3T3-E1 cells, and the expression of osteogenic markers significantly decreased. Integrin and downstream RhoA, FAK, and vinculin form an adhesive plaque complex and connect with F-actin. By changing the polymerization of F-actin and the proportion of monomers, the activation state of the downstream pathway can be changed (Arnsdorf et al., 2009; Tong et al., 2020). The second kind of mechanoreceptor molecules is a variety of ion channels found on the cell surface. Piezo1 is a newly discovered mechanoreceptor cation channel in recent years. It has been reported that Piezo1 is closely related to osteogenic differentiation. Since our team has discussed the role of F-actin in the differentiation of BMSCs in previous work (Tong et al., 2020), the relevant contents are not involved in this study.

The Yap/Taz pathway is a widely recognized mechanical response pathway. After receiving a mechanical signal, Yap and Taz will dephosphorylate and enter the nucleus to activate the downstream TEAD transcription factors and promote the transcription of related genes. Aragona et al. (Aragona et al., 2013) found that actin depolymerization-related molecule cofilin can regulate Yap/Taz pathway, suggesting the significance of the Yap/Taz pathway in the process of actin transmitting mechanical signal. At the same time, Tong et al. (Tong et al., 2020) also showed that the Yap/Taz pathway was a possible downstream signal pathway of F-actin. Therefore, this study focuses on the role of Yap in nanotube-stimulated osteogenesis. Contradictions exist in the previous literature study. In the research by Zhang et al.(Zhang et al., 2016), the authors believe that the activation of Yap is inhibited in the process of nanotube-promoting osteogenesis. In our study, Yap inhibitors and Yap knockdown/overexpression cell lines were used to prove that Yap activation plays a promoting role in both nanotube-stimulated osteogenesis and normal osteogenesis. However, the specific effector molecules downstream of Yap remain to be revealed. Verteporfin is a photosensitizer used in photodynamic therapy to eliminate abnormal intraocular blood vessels associated with age-related macular degeneration and other diseases (Brown et al., 2006). It can inhibit the combination of Yap and TEAD. Our data show that it can significantly inhibit the osteogenic differentiation of stem cells.

As mentioned earlier, Piezo1 has received extensive attention in recent years (Sugimoto et al., 2017). In recent years, many literature studies have reported its role in mechanical signal transduction and bone metabolism (Wang et al., 2020; Lee et al., 2021). Li et al. (Li et al., 2019) found administration of a Piezo1 agonist could increase bone mass, which mimicked the effect of physical exercise. In addition, conditional knockout of Piezo1 in osteoblasts and osteocytes greatly reduced bone strength in mice. Sugimoto et al. (Sugimoto et al., 2017) studied the role of Piezo1 in differentiation and proved its promoting effect on osteogenic differentiation. These studies all revealed the importance of Piezo1 in mechano-related osteogenesis. Studies by Japanese scholars (Hasegawa et al., 2021) have shown that Yap can regulate the expression of Piezo1 in oral squamous cell carcinoma cells and further regulate the proliferation and metastasis of squamous cell carcinoma. Our data shows that inhibiting Yap or knockdown and overexpression of Yap in osteoblasts will affect the expression of Piezo1 mRNA and protein. Therefore, our study preliminarily proved the possible downstream effector molecule, Piezo1, of Yap. However, whether such regulation is at the protein level or transcriptional level and how to regulate the expression of Piezo1 still need further research.

In conclusion, this study not only confirmed the important role of Yap in the process of nanotopography-promoting osteogenesis but also creatively proposed and verified its downstream effector molecule, Piezo1, a mechanosensitive cation channel.

## Data Availability

The original contributions presented in the study are included in the article/Supplementary Material; further inquiries can be directed to the corresponding authors.

## Abbreviations

ALP, alkaline phosphatase; BCA, bicinchoninic acid; BMSCs, bone marrow-derived mesenchymal stem cells; DMSO, dimethyl sulfoxide; EDS, X-ray energy dispersive analysis; FBS, fetal bovine serum; OM, osteogenic induction medium; PVDF, polyvinylidene fluoride; SEM, scanning electron microscopy; TBST, TBS + Tween; VP, verteporfin.

## References

[B1] Aragona M. Panciera T. Manfrin A. Giulitti S. Michielin F. Elvassore N. (2013). A Mechanical Checkpoint Controls Multicellular Growth through YAP/TAZ Regulation by Actin-Processing Factors. Cell 154 (5), 1047–1059. 10.1016/j.cell.2013.07.042 23954413

[B2] Arnsdorf E. J. Tummala P. Kwon R. Y. Jacobs C. R. (2009). Mechanically Induced Osteogenic Differentiation - the Role of RhoA, ROCKII and Cytoskeletal Dynamics. J. Cel. Sci. 122 (Pt 4), 546–553. 10.1242/jcs.036293 PMC2714434 19174467

[B3] Brown D. M. Kaiser P. K. Michels M. Soubrane G. Heier J. S. Kim R. Y. (2006). Ranibizumab versus Verteporfin for Neovascular Age-Related Macular Degeneration. N. Engl. J. Med. 355 (14), 1432–1444. 10.1056/NEJMoa062655 17021319

[B4] Cha B. H. Kim J. S. Bello A. Lee G. H. Kim D. H. Kim B. J. (2020). Efficient Isolation and Enrichment of Mesenchymal Stem Cells from Human Embryonic Stem Cells by Utilizing the Interaction between Integrin α 5 β 1 and Fibronectin. Adv. Sci. 7 (17), 2001365. 10.1002/advs.202001365 PMC7507081 32995130

[B5] Chang Y. Shao Y. Liu Y. Xia R. Tong Z. Zhang J. (2019). Mechanical Strain Promotes Osteogenic Differentiation of Mesenchymal Stem Cells on TiO2 Nanotubes Substrate. Biochem. Biophys. Res. Commun. 511 (4), 840–846. 10.1016/j.bbrc.2019.02.145 30850158

[B6] Curran J. M. Chen R. Hunt J. A. (2006). The Guidance of Human Mesenchymal Stem Cell Differentiation *In Vitro* by Controlled Modifications to the Cell Substrate. Biomaterials 27 (27), 4783–4793. 10.1016/j.biomaterials.2006.05.001 16735063

[B7] Deng Y. Lu J. Li W. Wu A. Zhang X. Tong W. (2018). Reciprocal Inhibition of YAP/TAZ and NF-κB Regulates Osteoarthritic Cartilage Degradation. Nat. Commun. 9 (1), 4564. 10.1038/s41467-018-07022-2 30385786 PMC6212432

[B8] Du Y. Montoya C. Orrego S. Wei X. Ling J. Lelkes P. I. (2019). Topographic Cues of a Novel Bilayered Scaffold Modulate Dental Pulp Stem Cells Differentiation by Regulating YAP Signalling through Cytoskeleton Adjustments. Cell Prolif. 52 (6), e12676. 10.1111/cpr.12676 31424140 PMC6869304

[B9] Dupont S. Morsut L. Aragona M. Enzo E. Giulitti S. Cordenonsi M. (2011). Role of YAP/TAZ in Mechanotransduction. Nature 474 (7350), 179–183. 10.1038/nature10137 21654799

[B10] Fan Y. Du Z. Ding Q. Zhang J. Op Den Winkel M. Gerbes A. (2021). SEPT6 Drives Hepatocellular Carcinoma Cell Proliferation, Migration and Invasion via the Hippo/YAP Signaling Pathway. Int. J. Oncol. 58 (6), 25. 10.3892/ijo.2021.5205 33846777 PMC8025964

[B11] Hao L. Li L. Wang P. Wang Z. Shi X. Guo M. (2019). Synergistic Osteogenesis Promoted by Magnetically Actuated Nano-Mechanical Stimuli. Nanoscale 11, 23423–23437. 10.1039/c9nr07170a 31799540

[B12] Hasegawa K. Fujii S. Matsumoto S. Tajiri Y. Kikuchi A. Kiyoshima T. (2021). YAP Signaling Induces PIEZO1 to Promote Oral Squamous Cell Carcinoma Cell Proliferation. J. Pathol. 253 (1), 80–93. 10.1002/path.5553 32985688

[B13] Kahlenberg C. A. Swarup I. Krell E. C. Heinz N. Figgie M. P. (2019). Causes of Revision in Young Patients Undergoing Total Hip Arthroplasty. J. Arthroplasty 34 (7), 1435–1440. 10.1016/j.arth.2019.03.014 30948287

[B14] Kelmer G. Stone A. H. Turcotte J. King P. J. (2021). Reasons for Revision: Primary Total Hip Arthroplasty Mechanisms of Failure. J. Am. Acad. Orthop. Surg. 29 (2), 78–87. 10.5435/jaaos-d-19-00860 32404682

[B15] Lee W. Nims R. J. Savadipour A. Zhang Q. Leddy H. A. Liu F. (2021). Inflammatory Signaling Sensitizes Piezo1 Mechanotransduction in Articular Chondrocytes as a Pathogenic Feed-Forward Mechanism in Osteoarthritis. Proc. Natl. Acad. Sci. U.S.A. 118 (13), e2001611118. 10.1073/pnas.2001611118 33758095 PMC8020656

[B16] Li X. Han L. Nookaew I. Mannen E. Silva M. J. Almeida M. (2019). Stimulation of Piezo1 by Mechanical Signals Promotes Bone Anabolism. Elife 8, e49631. 10.7554/eLife.49631 31588901 PMC6779475

[B17] Liu Y. Rath B. Tingart M. Eschweiler J. (2020). Role of Implants Surface Modification in Osseointegration: A Systematic Review. J. Biomed. Mater. Res. 108 (3), 470–484. 10.1002/jbm.a.36829 31664764

[B18] Lopes H. B. Freitas G. P. Fantacini D. M. C. Picanço‐Castro V. Covas D. T. Rosa A. L. (2019). Titanium with Nanotopography Induces Osteoblast Differentiation through Regulation of Integrin αV. J. Cel. Biochem. 120 (10), 16723–16732. 10.1002/jcb.28930 31090958

[B19] Lorthongpanich C. Thumanu K. Tangkiettrakul K. Jiamvoraphong N. Laowtammathron C. Damkham N. U-pratya Y. (2019). YAP as a Key Regulator of Adipo-Osteogenic Differentiation in Human MSCs. Stem Cel Res Ther. 10 (1), 402. 10.1186/s13287-019-1494-4 PMC6921580 31852542

[B20] Park J. Bauer S. von der Mark K. Schmuki P. (2007). Nanosize and Vitality: TiO2 Nanotube Diameter Directs Cell Fate. Nano Lett. 7 (6), 1686–1691. 10.1021/nl070678d 17503870

[B21] Park J. S. Kim M. Song N.-J. Kim J.-H. Seo D. Lee J.-H. (2019). A Reciprocal Role of the Smad4-Taz Axis in Osteogenesis and Adipogenesis of Mesenchymal Stem Cells. Stem Cells 37 (3), 368–381. 10.1002/stem.2949 30444564 PMC7379966

[B22] Perestrelo T. Correia M. Ramalho-Santos J. Wirtz D. (2018). Metabolic and Mechanical Cues Regulating Pluripotent Stem Cell Fate. Trends Cel. Biol. 28 (12), 1014–1029. 10.1016/j.tcb.2018.09.005 30361056

[B23] Reznikov N. Bilton M. Lari L. Stevens M. M. Kröger R. (2018). Fractal-like Hierarchical Organization of Bone Begins at the Nanoscale. Science 360 (6388), eaao2189. 10.1126/science.aao2189 29724924 PMC6037297

[B24] Schwartz B. E. Piponov H. I. Helder C. W. Mayers W. F. Gonzalez M. H. (2016). Revision Total Hip Arthroplasty in the United States: National Trends and In-Hospital Outcomes. Int. Orthop. (Sicot) 40 (9), 1793–1802. 10.1007/s00264-016-3121-7 26830782

[B25] Shah F. A. Thomsen P. Palmquist A. (2019). Osseointegration and Current Interpretations of the Bone-Implant Interface. Acta Biomater. 84, 1–15. 10.1016/j.actbio.2018.11.018 30445157

[B26] Shen B. Vardy K. Hughes P. Tasdogan A. Zhao Z. Yue R. (2019). Integrin Alpha11 Is an Osteolectin Receptor and Is Required for the Maintenance of Adult Skeletal Bone Mass. Elife 8, e42274. 10.7554/eLife.42274 30632962 PMC6349404

[B27] Shi Y. Li H. Zhang X. Fu Y. Huang Y. Lui P. P. Y. (2011). Continuous Cyclic Mechanical Tension Inhibited Runx2 Expression in Mesenchymal Stem Cells through RhoA-ERK1/2 Pathway. J. Cel. Physiol. 226 (8), 2159–2169. 10.1002/jcp.22551 21520068

[B28] Shi Y. Fu Y. Tong W. Geng Y. Lui P. P. Y. Tang T. (2012). Uniaxial Mechanical Tension Promoted Osteogenic Differentiation of Rat Tendon-Derived Stem Cells (rTDSCs) via the Wnt5a-RhoA Pathway. J. Cel. Biochem. 113 (10), 3133–3142. 10.1002/jcb.24190 22615126

[B29] Sugimoto A. Miyazaki A. Kawarabayashi K. Shono M. Akazawa Y. Hasegawa T. (2017). Piezo Type Mechanosensitive Ion Channel Component 1 Functions as a Regulator of the Cell Fate Determination of Mesenchymal Stem Cells. Sci. Rep. 7 (1), 17696. 10.1038/s41598-017-18089-0 29255201 PMC5735093

[B30] Sun Y. Villa-Diaz L. G. Lam R. H. W. Chen W. Krebsbach P. H. Fu J. (2012). Mechanics Regulates Fate Decisions of Human Embryonic Stem Cells. PLoS One 7 (5), e37178. 10.1371/journal.pone.0037178 22615930 PMC3353896

[B31] Tong Z. Liu Y. Xia R. Chang Y. Hu Y. Liu P. (2020). F-actin Regulates Osteoblastic Differentiation of Mesenchymal Stem Cells on TiO2 Nanotubes through MKL1 and YAP/TAZ. Nanoscale Res. Lett. 15 (1), 183. 10.1186/s11671-020-03415-9 32965618 PMC7511505

[B32] Wang N. Li H. Lü W. Li J. Wang J. Zhang Z. (2011). Effects of TiO2 Nanotubes with Different Diameters on Gene Expression and Osseointegration of Implants in Minipigs. Biomaterials 32 (29), 6900–6911. 10.1016/j.biomaterials.2011.06.023 21733571

[B33] Wang W. Liu Q. Zhang Y. Zhao L. (2014). Involvement of ILK/ERK1/2 and ILK/p38 Pathways in Mediating the Enhanced Osteoblast Differentiation by Micro/nanotopography. Acta Biomater. 10 (8), 3705–3715. 10.1016/j.actbio.2014.04.019 24769109

[B34] Wang L. You X. Lotinun S. Zhang L. Wu N. Zou W. (2020). Mechanical Sensing Protein PIEZO1 Regulates Bone Homeostasis via Osteoblast-Osteoclast Crosstalk. Nat. Commun. 11 (1), 282. 10.1038/s41467-019-14146-6 31941964 PMC6962448

[B35] Wu J. Lewis A. H. Grandl J. (2017). Touch, Tension, and Transduction - the Function and Regulation of Piezo Ion Channels. Trends Biochem. Sci. 42 (1), 57–71. 10.1016/j.tibs.2016.09.004 27743844 PMC5407468

[B36] Yang W. Lu X. Zhang T. Han W. Li J. He W. (2021). TAZ Inhibits Osteoclastogenesis by Attenuating TAK1/NF-κB Signaling. Bone Res. 9 (1), 33. Article 33. 10.1038/s41413-021-00151-3 34253712 PMC8275679

[B37] Yong K. W. Choi J. R. Choi J. Y. Cowie A. C. (2020). Recent Advances in Mechanically Loaded Human Mesenchymal Stem Cells for Bone Tissue Engineering. Ijms 21 (16), 5816. 10.3390/ijms21165816 PMC7461207 32823645

[B38] Zhang H. Cooper L. F. Zhang X. Zhang Y. Deng F. Song J. (2016). Titanium Nanotubes Induce Osteogenic Differentiation through the FAK/RhoA/YAP cascade. RSC Adv. 6 (50), 44062–44069. 10.1039/c6ra04002k

[B39] Zhang D. Ni N. Wang Y. Tang Z. Gao H. Ju Y. (2021). CircRNA-vgll3 Promotes Osteogenic Differentiation of Adipose-Derived Mesenchymal Stem Cells via Modulating miRNA-dependent Integrin α5 Expression. Cell Death Differ. 28 (1), 283–302. 10.1038/s41418-020-0600-6 32814879 PMC7853044

