# Supplementary material for: TiO2 Nanotubes Promote Osteogenic Differentiation Through Regulation of Yap and Piezo1
Source: Front Bioeng Biotechnol. 2022 Apr 7;10:872088. doi: 10.3389/fbioe.2022.872088 (PMC9023332; doi:10.3389/fbioe.2022.872088)
Supplement: Supplementary file 1 [file Table1.DOCX]

Table 1 Primers used in the qRT-PCR assay

| **Gene** | **Organisms** | **Forward (5′-3′)** | **Reverse (5′-3′)** |
| --- | --- | --- | --- |
| Gapdh | Rattus norvegicus | GGCAAGTTCAACGGCACAG | CGCCAGTAGACTCCACGACAT |
| Col1a1 | Rattus norvegicus | TGATGGACCTGCTGGCTCTC | GACCACGTTCACCACTTGCT |
| Osx | Rattus norvegicus | CCAATGACTACCCACCCTTTCC | ATGGATGCCCGCCTTGTA |
| Ocn | Rattus norvegicus | GGACCCTCTCTCTGCTCACTCTG | ACCTTACTGCCCTCCTGCTTGG |
| Opn | Rattus norvegicus | TGATGACGACGACGATGACGAC | TGTGCTGGCAGTGAAGGACTC |
| Alpl | Rattus norvegicus | GACAATGAGATGCCGCCAGAG | CATCCAGTTCATATTCCACATCAGTTC |
| Gapdh | Mus musculus | GGCAAGTTCAACGGCACAG | CGCCAGTAGACTCCACGACAT |
| Runx2 | Mus musculus | AGACCAGCAGCACTCCATATCTCT | CGTCAGCGTCAACACCATCATTCT |
| Osx | Mus musculus | AAGTTCACCTGCCTGCTCTGTTC | GGCGGCTGATTGGCTTCTTCTT |
| Ocn | Mus musculus | AAGCAGGAGGGCAATAAGGTAGTG | TCTTCAAGCCATACTGGTCTGATAGC |
| Opn | Mus musculus | GACGATGATGATGACGATGGAGACC | CTGTAGGGACGATTGGAGTGAAAGTG |
| Alpl | Mus musculus | TCACGGCGTCCATGAGCAGAA | TACAGGCAAGGCAGATAGCGAACT |
| Ctgf | Mus musculus | ACACCGCACAGAACCACCACTC | TAATGGCAGGCACAGGTCTTGATGAAC |
| Cyr61 | Mus musculus | ATACTGCGGCTCCTGCGTAG | CCTGAACTTGTGGATGTCATTGAATAG |
| Axl | Mus musculus | CTTGTGTCCATTCAACTGTGCTACG | TTCCATCCTCTTGCCGCTCAG |
| Yap | Mus musculus | GCCTACACTGGAGCAGGATGGA | GATAGGTGCCACTGTTAAGAAAGGGAT |
| Piezo1 | Mus musculus | AGTATCTGCTTCTTCTTCCTGCTCTTG | GACTTCTCCTCAATCTGGCGATGG |
