# Supplementary material for: TiO2 Nanotubes Promote Osteogenic Differentiation Through Regulation of Yap and Piezo1
Source: Front Bioeng Biotechnol. 2022 Apr 7;10:872088. doi: 10.3389/fbioe.2022.872088 (PMC9023332; doi:10.3389/fbioe.2022.872088)
Supplement: Supplementary file 2 [file DataSheet1.DOCX]

https://www.jianguoyun.com/p/DdN4bbEQ1JugChj_-asE
